# Unsupervised machine learning models reveal predictive clinical markers of glioblastoma patient survival using white blood cell counts prior to initiating chemoradiation

**DOI:** 10.1093/noajnl/vdad140

**Published:** 2023-11-11

**Authors:** Wesley Wang, Zeynep Temerit Kumm, Cindy Ho, Ideli Zanesco-Fontes, Gustavo Texiera, Rui Manuel Reis, Horacio Martinetto, Javaria Khan, Martin G McCandless, Katherine E Baker, Mark D Anderson, Muhammad Omar Chohan, Sasha Beyer, J Brad Elder, Pierre Giglio, José Javier Otero

**Affiliations:** Department of Pathology, Ohio State University Wexner Medical Center, Columbus, Ohio, USA; Department of Pathology, Ohio State University Wexner Medical Center, Columbus, Ohio, USA; Department of Pathology, Ohio State University Wexner Medical Center, Columbus, Ohio, USA; Molecular Oncology Research Center, Barretos Cancer Hospital, Barretos, Brazil; Department of Pathology, Barretos Cancer Hospital, Barretos, Brazil; Molecular Oncology Research Center, Barretos Cancer Hospital, Barretos, Brazil; Life and Health Sciences Research Institute (ICVS)/School of Medicine, University of Minho, Braga, Portugal; ICVS/3B’s—PT Government Associate Laboratory, Braga-Guimarães, Portugal; Departamento de Neuropatología y Biología Molecular, Instituto de Investigaciones Neurológicas Dr Raúl Carrea (FLENI), Buenos Aires, Argentina; Department of Pathology, University of Mississippi Medical Center, Jackson, Mississippi, USA; Department of Neuro-Oncology, University of Mississippi Medical Center, Jackson, Mississippi, USA; Department of Neurosurgery, University of Mississippi Medical Center, Jackson, Mississippi, USA; Department of Neuro-Oncology, University of Mississippi Medical Center, Jackson, Mississippi, USA; Department of Neurosurgery, University of Mississippi Medical Center, Jackson, Mississippi, USA; Department of Neuro-Oncology, University of Mississippi Medical Center, Jackson, Mississippi, USA; Department of Neurosurgery, University of Mississippi Medical Center, Jackson, Mississippi, USA; Department of Radiation Oncology, Ohio State University Wexner Medical Center, Columbus, Ohio, USA; Department of Neurosurgery, Ohio State University Wexner Medical Center, Columbus, Ohio, USA; Department of Neurology, Ohio State University Wexner Medical Center, Columbus, Ohio, USA; Department of Pathology, Ohio State University Wexner Medical Center, Columbus, Ohio, USA

**Keywords:** clinical decision-making, glioblastoma, machine learning, survival outcomes

## Abstract

**Background:**

Glioblastoma is a malignant brain tumor requiring careful clinical monitoring even after primary management. Personalized medicine has suggested the use of various molecular biomarkers as predictors of patient prognosis or factors utilized for clinical decision-making. However, the accessibility of such molecular testing poses a constraint for various institutes requiring identification of low-cost predictive biomarkers to ensure equitable care.

**Methods:**

We collected retrospective data from patients seen at Ohio State University, University of Mississippi, Barretos Cancer Hospital (Brazil), and FLENI (Argentina) who were managed for glioblastoma—amounting to 581 patient records documented using REDCap. Patients were evaluated using an unsupervised machine learning approach comprised of dimensionality reduction and eigenvector analysis to visualize the inter-relationship of collected clinical features.

**Results:**

We discovered that the serum white blood cell (WBC) count of a patient during baseline planning for treatment was predictive of overall survival with an over 6-month median survival difference between the upper and lower quartiles of WBC count. By utilizing an objective PD-L1 immunohistochemistry quantification algorithm, we were further able to identify an increase in PD-L1 expression in glioblastoma patients with high serum WBC counts.

**Conclusions:**

These findings suggest that in a subset of glioblastoma patients the incorporation of WBC count and PD-L1 expression in the brain tumor biopsy as simple biomarkers predicting glioblastoma patient survival. Moreover, machine learning models allow the distillation of complex clinical data sets to uncover novel and meaningful clinical relationships.

**Key Points**

- Unsupervised learning models visualize complex data sets to uncover novel relationships.
- White blood cell count and PD-L1 expression are simple biomarkers predictive of glioblastoma patient survival.

**Importance of the Study**

Glioblastomas remain a challenging malignancy to manage following surgical resection and chemoradiation. Efforts to identify prognostic markers of disease are therefore critical in ascertaining how a provider will manage a patient’s tumor. Current efforts have largely focused on molecular markers to stratify patient populations, but these approaches are not equally accessible. Building upon studies that have evaluated routinely collected clinical data, we highlight the use of machine learning-based approaches to rapidly visualize relationships in clinical data to uncover novel trends. Moreover, the use of pretreatment blood count may play a role in patient prognostication and tumor microenvironment status. These findings thus highlight the importance of future exploration of patient tumor-immune activity while lowering the complexity of assessment through machine learning.

Glioblastoma (GB) patients suffer from aggressive solid tumors of the central nervous system, with 95% projected to be deceased within 5 years following diagnosis.^1^ Cause of death in glioblastoma varies but may include herniation secondary to mass effect, treatment complications, and aspiration pneumonia due to brainstem dysfunction.^2^ Despite aggressive adjuvant therapy, however, almost all patients will experience tumor progression. To address this, major strides have been made within neuro-oncology to merge personalized medicine to better predict patient outcomes. Although therapies for GB management after maximal-safe surgical resection have remained largely unchanged since the concomitant use of temozolomide (TMZ) with radiation (ChemoRT), molecular biomarkers have been integrated as critical tools in the diagnosis and prognostication of gliomas.^3,4^ From a diagnostic standpoint, the lack of *IDH* mutation defines the current World Health Organization (WHO) 2021 definition of GB.^4^ Moreover, *TERT* promoter mutations, EGFR amplifications, and modifications of chromosome 7/9/10 are all recognized molecular changes present in GB.

Prognostically, several molecular markers have been suggestive of predicting outcomes in GB patients. Namely, *MGMT* promoter methylation status is routinely tested to predict TMZ efficacy due to the antagonistic role of MGMT-mediated repair following TMZ-induced DNA alkylation.^5,6^ However, other markers such as CDKN2A/B loss and EGFRvIII have been shown in retrospective studies to act as poor prognostic markers in GB patients or subset populations.^7,8^ The need for prognostic markers of disease is critical in ascertaining whether certain patients should be monitored more closely during follow-up. Furthermore, gold standard management of GB requires aggressive multimodal treatment amongst neuro-oncology, neurosurgery, neuroradiology, and radiation oncology which would largely benefit from improved triaging methods that prioritize which patients should or should not have such care. Moreso, precise prognostic grading benefits patients and families when discussing management direction.

Unfortunately, molecular marker accessibility is not equal across healthcare ecosystems.^9,10^ Current discussions in the field of neuro-oncology have pointed to major accessibility barriers and bioethical implications of pure reliance on molecular biomarkers of disease to understand cancer.^10^ Molecular pathology testing requires batching to reduce patient costs, resulting in significant turn-around time delays for molecular assays.^11^ In consequence, groups have sought ways to evaluate outcomes of glioma patients using surrogate measures such as neuro-cognitive testing, psychiatric examination, or image analysis of histology to predict molecular phenotypes as these turn-around-times are superior to those of molecular pathology.^9,12,13^ Although promising, however, integration of these approaches will take time whereas routinely collected data is readily available.

To better explore potential prognostic markers already routinely collected while managing GB patients, we retrospectively evaluated 581 patients at 4 sites from 3 countries: Ohio State University (USA), University of Mississippi (USA), Barretos Cancer Hospital (Brazil), and FLENI (Argentina). Patient features were assessed using an unsupervised learning approach to understand how clinical metrics related to each other—including relevant clinical endpoints such as progression-free survival (PFS) and overall survival (OS). Furthermore, by utilizing these novel relationships, we better characterized the relevance of routine complete blood counts (CBCs) and merged its utility with IHC staining in pathology to suggest novel workflows that may predict outcomes of patients with GB.

## Materials and Methods

### Selection Criteria and Clinical Data Collection

Evaluation of retrospective clinical charts from the electronic health record (EHR) was performed at the Ohio State University (OSU) with IRB approval under study number 2020C0062, University of Mississippi (UMMC) with IRB approval under study number UMMC-IRB-2022-93, Barretos Cancer Hospital with IRB approval under study number 1604/2018, and FLENI under ethics committee/patient’s informed consent approval. Clinical records from patients receiving GB care from 2012 to 2020 were evaluated. Inclusion criteria for patients were designated by prior history of GB treatment with total surgical resection and ChemoRT. Following WHO 2021 guidelines, assessed GBs were defined using Grade-4 pathology and confirmation of *IDH* wild-type status.^4^ Prior histories of low-grade gliomas, *IDH* mutation, or patients with poor documentation of disease course were excluded. Study data were collected and managed using REDCap electronic data capture tools.^14,15^ REDCap was utilized to (a) securely store and deidentify patient records for downstream use, (b) ensure consistency of data collection, and (c) be distributable to collaborators desiring to replicate or collect similar metrics as described in the study (Supplementary Document 1).

Clinical features were collected as listed in the EHR. Comorbidity scoring was performed by manually listing known comorbidities and scoring them in REDCap following the Charlson Comorbidity Index (CCI).^16^ Karnofsky performance scale (KPS) was recorded at the time of initial diagnosis to assess patient functional status. Lesion and molecular features were extracted from radiology and pathology reports, respectively. Results from CBCs—including white blood cell count (WBC), neutrophil count, lymphocyte count, and platelet count were designated as CBC draws occurring approximately 2–4 weeks after surgery during patient follow-up with neuro-oncology prior to beginning ChemoRT. The neutrophil-to-lymphocyte ratio (NLR) was calculated by dividing the neutrophil count by the lymphocyte count in a patient. Steroid dose was defined as the total daily dexamethasone steroid intake during the same day as CBC collection. Variables containing dates were converted to deidentified values of time to remove potential patient timeline identification after calculating relevant timespans in days. OS in the study was defined as the time from primary tumor resection until the time of death. PFS was defined as time from primary tumor resection until the time of initial detection of a novel enhancing lesion on imaging. Confirmation of enhancement as being either cancer-recurrent or treatment-reactive in nature was completed by clinical correlation and consensus from a multidisciplinary tumor board.

### Exploration of Clinical Features

Collected clinical data from REDCap was exported and deidentified for use in R. Missingness of data was evaluated using the naniar package.^17^ Cases with over 30% missing records were not evaluated using unsupervised analysis while any missing features were imputed using the multiple imputation by chained equations (mice) package in R in order to perform principal component analysis (PCA) visualization alone (Supplementary Figure 1).^18^ Using the factoextra package, PCA was performed over the data set and relations of clinical features were visualized using eigenvector plotting of our PCA as described in our previous work.^19,20^

Returning to our non-imputed data, the exploration of clinical features as a function of OS was explored using Cox regression modeling with a specific assessment of the relationships uncovered in our PCA visualization. Clinical features were independently evaluated using a univariate Cox model to assess the prediction of OS time. *P*-values less than .05 were omitted in subsequent modeling. Univariate-significant features were then evaluated using a multivariate Cox regression model using patient records found to have no missing relevant features. Patient survival was further explored by quartile stratification of patients for relevant clinical features (Supplementary Table 1). The highest and lowest 25% groups were compared for PFS and OS using the survival and survminer packages.^21,22^ Visualization of relevant clinical confounders was performed using R with ggplot2.^23^

### PD-L1 Image Analysis

PD-L1 immunohistochemistry imaging was collected from relevant patient samples at Ohio State which underwent routine PD-L1 evaluation at the time of primary surgery for GB. Image tiles were collected from digital pathology slides and processed in R using EBImage.^24^ RBG images were deconvoluted for hematoxylin and DAB stain layers using sci-kit-image and reticulate.^25,26^ Segmentation for either hematoxylin or DAB staining was performed using Otsu thresholding.^27^ Segmented regions were calculated for morphologic features using EBImage. Filtration of segmentation was performed using a random-forest-based classifier trained over intensity-based morphology features to classify segmentation as no stain, low stain, medium stain, and high stain (Supplementary Figure 2).^28^ No stain segmentations were filtered. PD-L1 staining was represented by the ratio of DAB-stained pixels to hematoxylin-stained pixels—calculated from segmentation areas in EBImage based upon objective stain scoring approaches delineated by Igarashi et al.^29^

## Results

### Patient Characteristics

**Figure 1.**
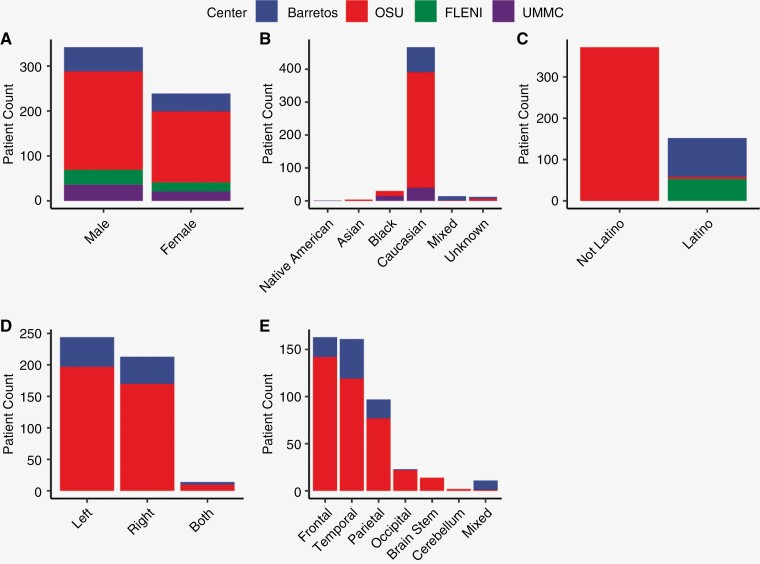
Distribution of patient demographics and lesion characteristics with respect to center. Bar plot distributions of (**A**) gender, (**B**) race, (**C**) ethnicity, (**D**) lesion sidedness, and (**E**) lesion lobe location across collected centers. Centers are represented as OSU (red), UMMC (purple), Barretos (blue), and FLENI (green). Omission of center in plot indicates variable was not collected.

A total of 581 patients were evaluated and documented in REDCap across 4 centers. In turn, overall demographic scores and biases across centers were assessed. Overall, the mean age was 61 years (range: 20–89; Supplementary Table 2). Men slightly exceeded woman in representation across centers (Figure 1A). The distribution of ethnic origin is directly related to the site of collection. North American sites represented predominately Caucasians and Black/African Americans with non-Latino ethnicity, while South American sites were of Caucasian and mixed ancestry—that in U.S. surveys would be considered Hispanic/Latino (Figure 1B and C). All patients had biopsy confirmed GB with total resection of tumor at primary surgery. There was a slight predominance of left-sided lesions primarily occurring in the frontal, temporal, and parietal lobes across sites (Figure 1D and E). On average, tumor diameter was 4.34 cm (range: 0.1–9.50 with most without midline shift on imaging with KPS near 80 (range: 30–100; Supplementary Table 2). It was however noted that several clinical features were not collected in sites outside OSU (Supplementary Figure 1). When tested, IHC studies showed primarily ATRX locus intact (98%) and p53 mutation (78%) as defined by positivity in over 10% of cells. Predominant molecular features were *EGFR* amplification (56%) and unmethylated *MGMT* promoter status (57%).

**Figure 2.**
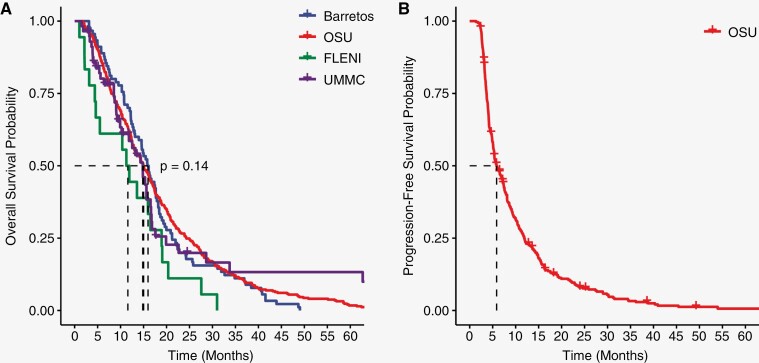
Overall survival and progression-free survival across sites. (**A**) OS and (**B**) PFS of patients across sites. Centers are represented as OSU (red), UMMC (purple), Barretos (blue), and FLENI (green).

ChemoRT overall followed a traditional 60 Gray-30 fractions radiation plan, but some patients received a hypo-fractionated regimen or did not complete ChemoRT (Radiation Plan: 55.59 [range: 5.34–75]; Radiation Fractions: 26.8 [range: 1–50]). Adjuvant TMZ therapy was completed at 3 cycles on average (range: 0–19). Symptomatic management of edema with steroids at the time of CBC collection was on average 2.84 mg/day with a broad range of use (range: 0–24). Outcomes of patients were assessed as both PFS and OS from the date of primary surgery. Among centers, the median OS ranged from 12 to 16 months without significant difference among groups while the median PFS was 6 months at OSU (Figure 2).

### Unsupervised Analysis of Patient Reveals Capabilities of CBCs in Predicting Overall Survival Outcome and Time to Enhancement

**Figure 3.**
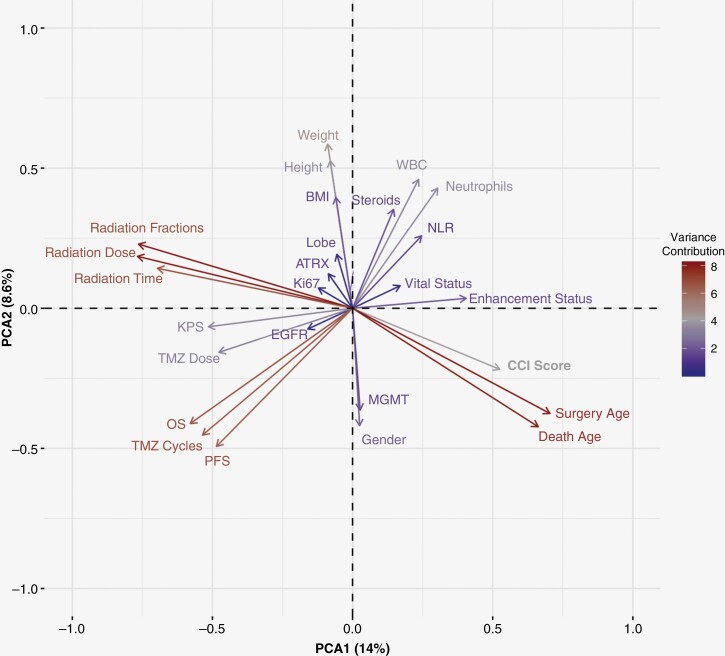
Eigenvector analysis of clinical features. Collected clinical features from Redcap data were assessed for all patients with less than 30% of data missing (*n* = 375). Missing data was imputed in R using multiple imputation by chained equations. Contribution to data variation is highlighted by the color legend and distance of a vector from the origin. Relationship of features are represented by directionality with direct relationships represented by same directionality, inverse relationship by opposite directionality, and no relationship as orthogonal directionality. Omitted features did not have high enough variance contribution to visualize.

Utilizing the various collected clinical data points, we sought to evaluate whether specific clinical features were correlated with relevant outcomes in patients that have not been previously integrated into clinical practice. Specifically, we posited that applying an unsupervised machine learning approach could reveal clinical relationships amongst features permitting us to visualize novel findings predictive in GB patient prognosis. To do so, our multidimensional data set was reduced using principal components analysis (PCA) to stratify patients by these clinical metrics and visualized using PCA eigenvector plotting (Figure 3). The directionality of eigenvectors (arrows) amongst other eigenvectors represents direct correlations through same arrow directionality, inverse correlations through opposite directionality, and no correlation through orthogonal directionality. With these considerations, we identified relationships of features to relevant clinical outcomes. Notably, the directionality of eigenvectors for OS and PFS occurred similarly [lower left quadrant] with inverse directionality to CBC-related measures—note that WBC and neutrophil measurements are in the upper right quadrant indicating that as WBC increased OS and PFS decreased (Figure 3). In contrast, enhancement status was shown on the right side of the plot, but other vectors had less robust directional relationships—with the most prominent being inverse directionality of KPS and adjuvant TMZ dosage in the left quadrant. Nevertheless, as our eigenvector plot uncovered novel variations amongst clinical features, we further explored these findings using regression modeling.

Cox regression modeling was applied to predict OS based on our clinical features. Based on our PCA eigenvector plot, we theorized that CBC-related metrics would be significant in predicting survival time. Univariate models were first performed over the study population to assess which features were found to have significance. In total, 12 separate features were found to be significant (Patient Age, KPS, CCI score, *MGMT* methylation status, WBC count, Neutrophil count, NLR, Radiation Dose, Radiation Fractions, Overall Radiation Time, Adjuvant TMZ Cycles, and Adjuvant TMZ Dose; Supplementary Table 3). A multivariate Cox regression model was constructed using 9 significant features from Supplementary Table 3 which were not derived from each other (ie, neutrophil load is represented within WBC load or overall radiation time is affected by the fractionation of radiation). Six clinical features were found to be relevant (Patient Age at surgery, KPS at diagnosis, *MGMT* methylation status, WBC count at follow-up, Total Radiation Dose, and Completed Adjuvant TMZ Cycles; Supplementary Table 4). Although the contribution of WBC count was significant in our multivariate model, the hazard ratio (HR) was small (1.023 [1.001–1.046]). In consequence, although a small, but significant risk to poorer survival was evidenced by increased WBC load, we next sought to explore the causes of the observed difference our Cox modeling showed against our PCA eigenvector plot.

**Figure 4.**
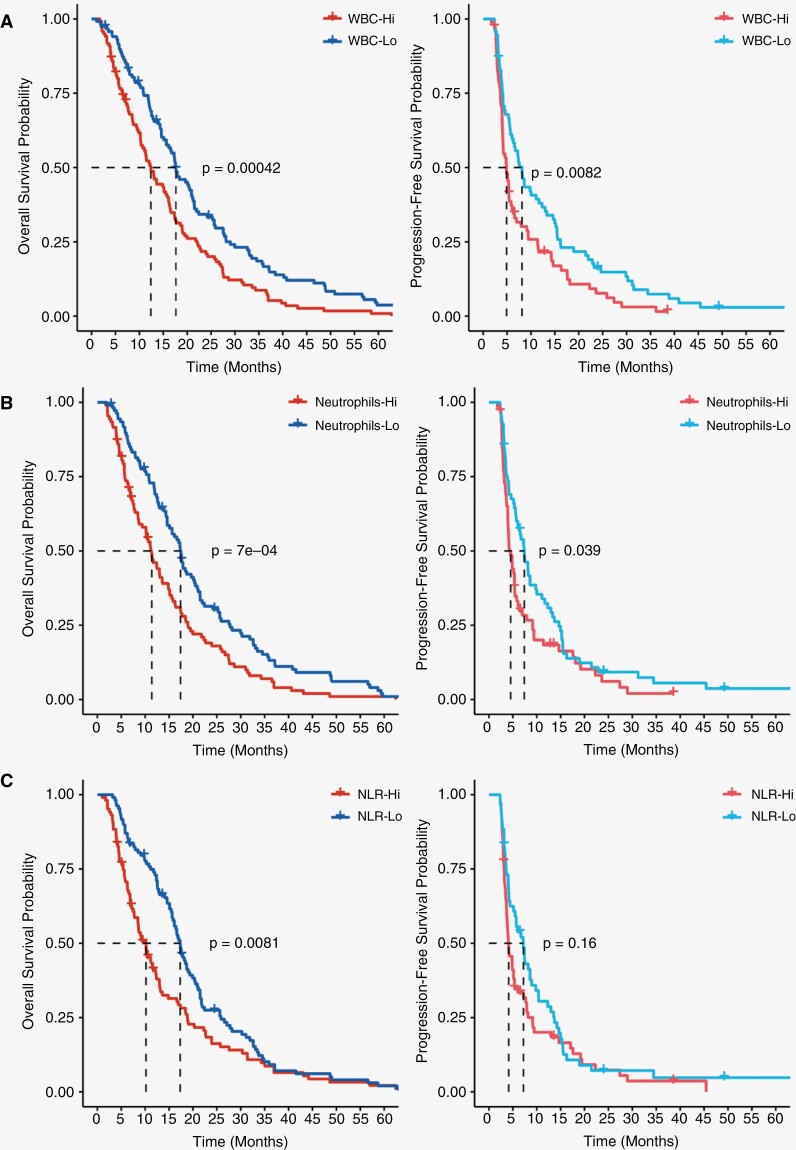
KM curve survival outcomes based upon CBCs. OS (left) and PFS (right) of patients in upper 25% [red] and lower 25% [blue] of cases stratified based on (**A**) WBC count (*n* = 186/group), (**B**) Neutrophil count (*n* = 212/group), and (**C**) Neutrophil-to-Lymphocyte ratio (*n* = 217/group). All CBCs were collected prior to initiating ChemoRT as routine baseline by neuro-oncology.

Although Cox regression modeling validated the finding that CBCs have predictive capabilities for survival time, we further examined the discordance of our strong correlations seen in the PCA eigenvector plot against the smaller HRs calculated in our Cox models. Specifically, we hypothesized the differences seen in our results may be underscored by robust survival differences present in the extremes of our CBC metrics. Specifically, based on our univariate Cox results, we explored survival differences in patients when stratified by WBC count, neutrophil count, and neutrophil:lymphocyte ratio. CBC measures were evaluated by stratifying populations into quartiles with the lower 25% (Lo in blue) and upper 25% (hi in red) of patients evaluated. In both overall WBC load and neutrophil load, PFS was significantly worse in the Hi group relative to the Lo group (*P* = .0082 and *P* = .039, respectively; Figure 4A and B). Furthermore, evaluation of OS using WBC load and neutrophils showed similar trends between groups (*P* = .00042 and *P* = .0007, respectively). However, while NLR did show a significant survival difference in OS (*P* = .0081), the difference in PFS between groups did not reach our threshold of significance (Figure 4C). Combined with the previous analyses, our findings strongly support the use of CBCs in predicting both OS and PFS in patients. Specifically, the evaluation of routinely drawn WBC and neutrophil count at the time of pre-ChemoRT planning is shown to be correlative with poorer survival outcomes in patients with high load compared to patients with low load.

### WBC Load is Reflective of Intrinsic Tumor Microenvironment Changes Present in Glioblastoma

**Figure 5.**
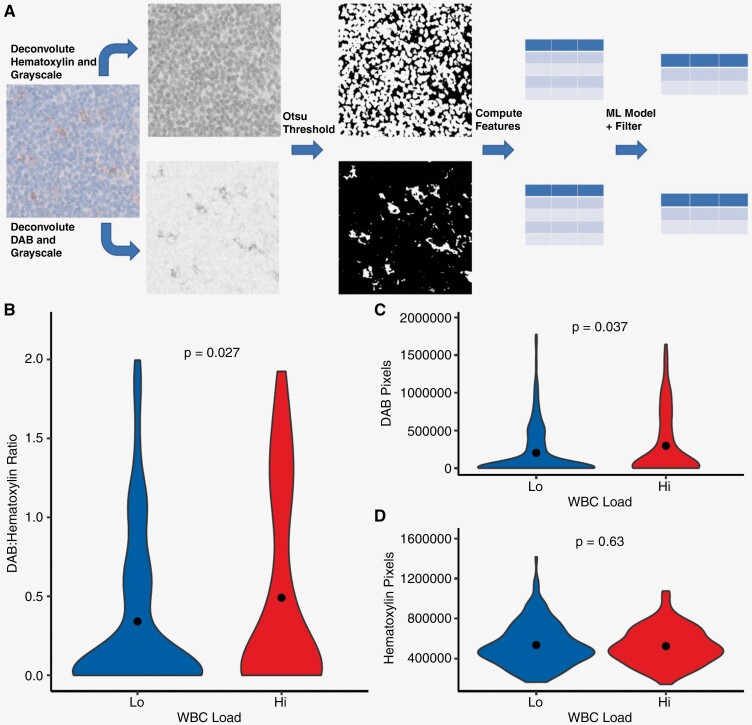
Assessment of PD-L1 expression in relation to WBC load. (**A**) Workflow for automatic PD-L1 signal quantification and morphology calculation. Violin plots of (**B**) DAB to Hematoxylin pixel ratio, (**C**) DAB pixel, and (**D**) Hematoxylin pixel distribution in WBC-Hi and WBC-Lo groups.

As the measures of WBC load evaluate circulating immune counts, we posited these differences in peripheral immune activity may correlate with intrinsic tumor microenvironment differences found in primary GB events. To assess this, we selected PD-L1 IHC staining done in a subset of patients during clinical evaluation. Amongst our study population, 57 cases had been evaluated by neuropathology for PD-L1 expression, and representative images were collected from cases and segmented using computer vision techniques to objectively quantify staining (Figure 5A). Staining was quantified as the ratio of DAB-positive PD-L1 staining against hematoxylin nuclear staining to control for tissue cellularity. In turn, increased detection of PD-L1 staining is represented by an increased DAB:hematoxylin ratio. It was observed that the WBC-Hi group showed higher ratios of PD-L1 DAB to hematoxylin staining when compared to WBC-Lo (*P* = .027, Figure 5B). Furthermore, assessing the 2 sides of the ratio comparison it was seen that while the amount of DAB pixels detected in an image was higher in the WBC-Hi group (*P* = .037), the detection of hematoxylin pixels did not vary between groups (*P* = 0.63; Figure 5C and D). In conclusion, the increased PD-L1 staining ratio in the WBC-Hi group was not a product of increased cellularity as the distribution of hematoxylin was not different. Overall, these findings indicate that an increase in PD-L1 staining was correlated with the WBC-Hi group which showed poorer survival outcomes in patients—validating that the observed differences in WBC load are correlative to initial immune activity present in GB lesions at resection.

### Steroid Tapering is Highly Heterogenous Following Surgery and May Influence WBC Load

**Figure 6.**
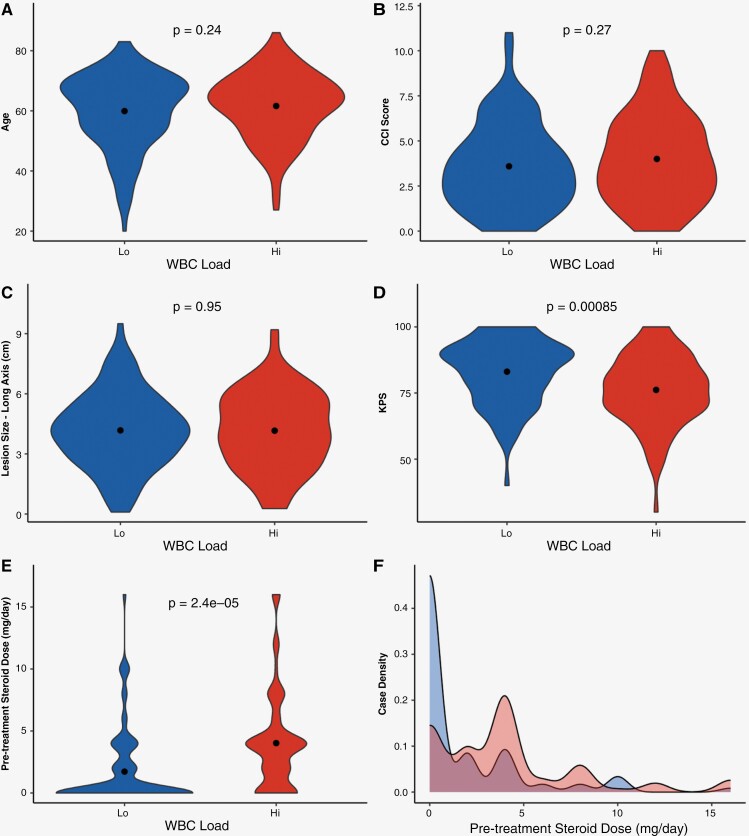
Functional status and steroid tapering vary amongst WBC-Hi/Lo groups. Groups were compared between (**A**) patient age, (**B**) CCI score, (**C**) lesion size, (**D**) KPS, and (**E**) steroid dosing at time of CBC collection using violin plots. (**F**) Distribution of daily dexamethasone steroid intake is visualized using a density plot between groups.

Although our analyses found a strong correlation between CBC load to survival outcomes, we further evaluated potential clinical confounders that may influence CBC levels prior to ChemoRT. As shown in the analyses of our study population, heterogeneity in patient demographics, lesion characteristics, and patient management was present (Supplementary Table 2). In turn, an assessment to identify whether specific clinical features significantly varied between our Hi and Lo populations was critical. As patient age, CCI score, KPS, and *MGMT* methylation status were predictive of survival in our univariate Cox model, we assessed these factors in addition to other clinical features that were shown to be correlative to CBC measures in the eigenvector plot (lesion size and steroid intake; Figure 3 and Supplementary Table 3). Comparing WBC-Hi and Lo groups, *MGMT* methylation status distribution was not found to significantly vary between groups (*X*^2^ = 0.88, *P*-value = .35). However, while patient age, CCI score, and lesion size were found to not vary between groups, significant variations in KPS (*P* = 8.5e-04) and steroid dosing (*P* = 2.4e-05) between groups were present with the WBC-Hi group showing a higher mean daily steroid intake compared to those in the Lo group (Figure 6A–E). Evaluating the distribution of steroid doses given to patients at the time of post-surgical CBC, a larger percentage of the patient from the WBC-Lo fully tapered off steroids (Hi: 24.2%; Lo: 65.1%). Nevertheless, an assay of patient distribution shows 55.9% of patients across both groups remain on steroids at follow-up prior to initiating ChemoRT (Figure 6 F). Assessment of survival between Hi and Lo groups in patients that fully tapered off steroids showed significant PFS differences when stratified by WBC load (*P*** = **.045) and neutrophil load (*P* = .026), but significant trends were not seen for OS (Supplementary Figure 3), Overall, these findings not only highlight the additional confounding role steroid intake and patient functional status may have on the survival differences seen between CBC load, but also emphasizes the heterogeneity of steroid dosing in patients following GB resection.

## Discussion

### PCA Eigenvector Visualization can Better Uncover Clinical Relationships in Complex Clinical Data Sets

To acknowledge, as shown in Supplementary Table 2, a large majority of features were missing amongst sites, thus findings should be viewed as a large single institution retrospective study with external validation needed in the future. With the adoption of the EHR, patient-related data has exponentially grown but remains an under-used resource due to the complexity of mining data and uncovering novel associations. Such data provides the potential for generating new patient stratification strategies.^30^ Our unsupervised PCA eigenvector approach may help rectify these challenges by providing easy-to-interpret visualizations of clinical data relationships. Illustrating this method’s utility in our study, the vectors measuring radiation metrics showed opposite directionality to features measuring patient age. The directionality of these vectors thus underscores the known clinical management of GB patients whereby older individuals often receive hypo-fractionated radiation dosage due to toxicity.^31^ The application of our eigenvector plot to initially detect relationships of patient survival to CBCs illustrates the utility of applying this method in large clinical data sets as a first-pass visualization approach in identifying novel relationships to explore in a clinical study.

### CBC Stratification can be Applied to Identify Patients with Poorer Predicted Survival Outcome

It was highlighted that CBC metrics—namely WBC and neutrophil count—were inversely related to OS and PFS. Interestingly, past studies have indicated that several components of CBC tests have predictive outcomes in OS. Namely, Pierscianek et al. and Jarmuzek et al. both retrospectively identified similar effects of WBC counts as prognostic factors in OS using CBCs collected during admission or pre-operatively for a potential glioma.^32,33^ Nevertheless, these studies do not recapitulate the relevant timepoint within our study which suggested WBC counts collected prior to initiating ChemoRT as the most predictive of survival. To this point, Schernberg et al. similarly assessed the utilization of CBCs during pretreatment for ChemoRT and found neutrophilia, advanced age, and more complete resection as features that independently decreased OS in a multivariate model, while steroid consumption did not.^34^ These findings largely parallel our Cox regression models which found patient age, ChemoRT treatment regime, and WBC count as predictive of OS. It was noted as well that the median survival time in the WBC-Hi group was approximately 12 months while the WBC-Lo group was 18 months. Although the coverage of patients in our study ranges over the past decade, studies have reported the median survival of GB ranging from 12 to 15 months with ChemoRT treatment.^35–37^ In turn, it may be suggestive that patients with lower WBC load experience better survival outcomes than just those with neutrophilia declining more rapidly. Conversely, assessing the cutoffs used for our WBC (Lo < 6.70; Hi > 11.90) and neutrophil (Lo < 4.40; Hi > 9.26) groupings, our low cutoffs are contained in the normal CBC reference range, but high cutoffs exceed the upper limit of normal,^38^ Thus patients with elevated WBC at ChemoRT planning may warrant more careful monitoring.

The relationship between survival and WBC count additionally raises applications to future therapeutic treatment of GBs- namely immunotherapies. Although effective in other solid tumors, the success of PD-1/PD-L1 immune checkpoint inhibitors and dendritic cell vaccines have been marginal in GB.^39–41^ Contrary to beliefs that GBs were largely immune-privileged tumors, growing evidence supports robust recruitment of pro-tumor-immune cell populations such as tumor-associated neutrophils and polymorphonuclear myeloid-derived suppressor cells.^42,43^ Thus, our poorly survived patients with elevated WBC/neutrophil load may underscore these biological mechanisms of tumor progression. Although future exploration is needed, such routine markers may be critical in identifying patients who are poor candidates for immunotherapy treatment due to unfavorable tumor microenvironment.

### Fast Tapering of Steroids following Surgery May Influence Long Term Outcomes of Patients

An important consideration that should be highlighted in our study was the evidence that prior to ChemoRT treatment, a large variance in steroid tapering and functional status (KPS) was noted across our study groups. Namely, while studies have evidenced that WBC prediction is independent of corticosteroid use statistically, the biological influence of corticosteroids on both neutrophil and lymphocyte count has been long recognized.^44,45^ Alternative studies, like Dubinski et al., have directly implicated that the administration of dexamethasone induces leukocytosis which was associated with poor survival.^46^ Conversely, however, as our study found poorer KPS in the WBC-Hi group, these differences in steroid dosing may reflect more advanced disease. Nevertheless, although steroids have been long used to provide supportive therapy, these findings may suggest the need to set a more consistent standard of quickly tapering patients off steroids or identifying who benefits from a prolonged course.^47^ Our observed WBC counts and PD-L1 measure may be suggestive of which patients in fact need to have steroid doses modified due to influence on immune cell activity. Studies have evidenced that the increased administration of steroids promotes immune cell dysfunction, namely in T-cell compartments, by promoting increased expression of PD-L1 in the microenvironment that advances dysregulation of the immune response.^48,49^ Although these findings may complicate the utilization of CBC measures for survival prediction, these observations, more importantly, underscore the need to better consider the use of steroids for the symptomatic relief of GB patients. Although steroids may be used for patients with worse functional status, steroids may conversely worsen symptoms by causing immune cell dysfunction.

## Conclusions

The use of data derived from the EHR will remain a powerful resource. However, the sheer complexity of both collecting and assaying data to uncover novel research discoveries poses a challenge for future studies. The deliverables of our study include our standardized REDCap data collection form for evaluation of GB, an unsupervised analysis framework to initially explore clinical data sets through PCA eigenvector visualization, and an automated image analysis pipeline for PD-L1 staining. Furthermore, the measure of CBC load at pretreatment for ChemoRT can be applied to identify patients at risk for unfavorable survival due to high WBC load coupled with elevated PD-L1 staining. However, deeper exploration and validation of these relationships and the tapering of steroids are important considerations in future studies of GB patient management.

## Supplementary Material

vdad140_suppl_Supplementary_Material

vdad140_suppl_Supplementary_Data

## Data Availability

The data sets used and/or analyzed during the current study are available from the corresponding authors upon reasonable request. Dissemination of the applied REDCap form can be found in supplement or supplied from corresponding authors.
