# Supplementary material for: Unsupervised machine learning models reveal predictive clinical markers of glioblastoma patient survival using white blood cell counts prior to initiating chemoradiation
Source: Neurooncol Adv. 2023 Nov 11;6(1):vdad140. doi: 10.1093/noajnl/vdad140 (PMC10894654; doi:10.1093/noajnl/vdad140)
Supplement: vdad140_suppl_Supplementary_Material [file vdad140_suppl_supplementary_material.docx]

**Supplementary Figures and Tables**

[Attached as Wang_et_al_Supplementary_Document_1.csv]

**Supplementary Document 1: REDCap Data Dictionary.** Variables unused in study were commonly missing in patients or shown to be non-significant in preliminary studies and

omitted. Data dictionary can be uploaded in REDCap in the dictionary tab to add tools.


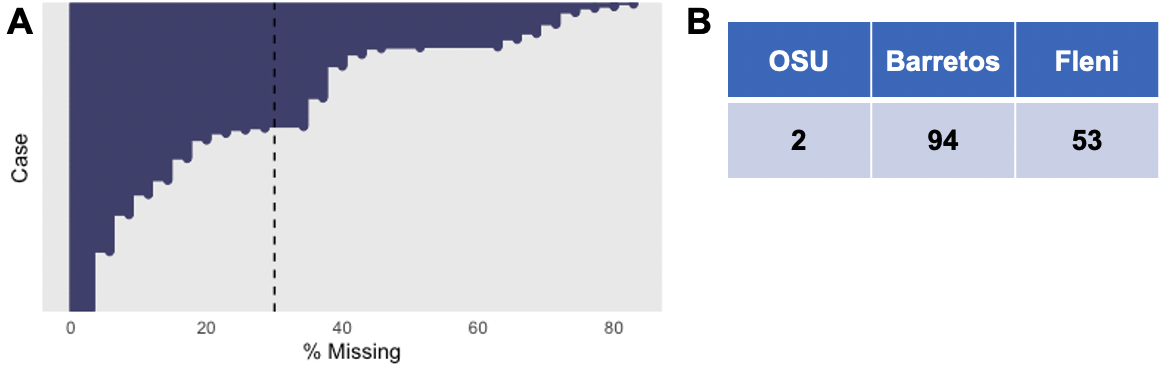


**Supplementary Figure 1: Missingness of cases for clinical features.** Distribution of clinical cases for percent missing clinical data is shown in (A). Cases with more than 30% missing data were omitted in the eigenvector feature plot due to needs of data imputation. Cutoff is shown in the dotted line with the distribution of cases omitted in table (B).

| **Count Measure** | **Lower Cut-off** | **Upper Cut-off** |
| --- | --- | --- |
| White Blood Cell (WBC) | 6.70 | 11.90 |
| Neutrophils | 4.40 | 9.26 |
| Neutrophil:Lymphocyte Ratio (NLR) | 2.58 | 8.35 |

**Supplementary Table 1: CBC cut-offs for quartile groupings.** Utilized cut-offs to define upper and lower 25% of patients in population. Cut-offs were calculated in R and set so that 25% of patients would be contained below or above the lower or upper cut-off respectively. CBC cutoffs are defined in (x10^9/L) units.


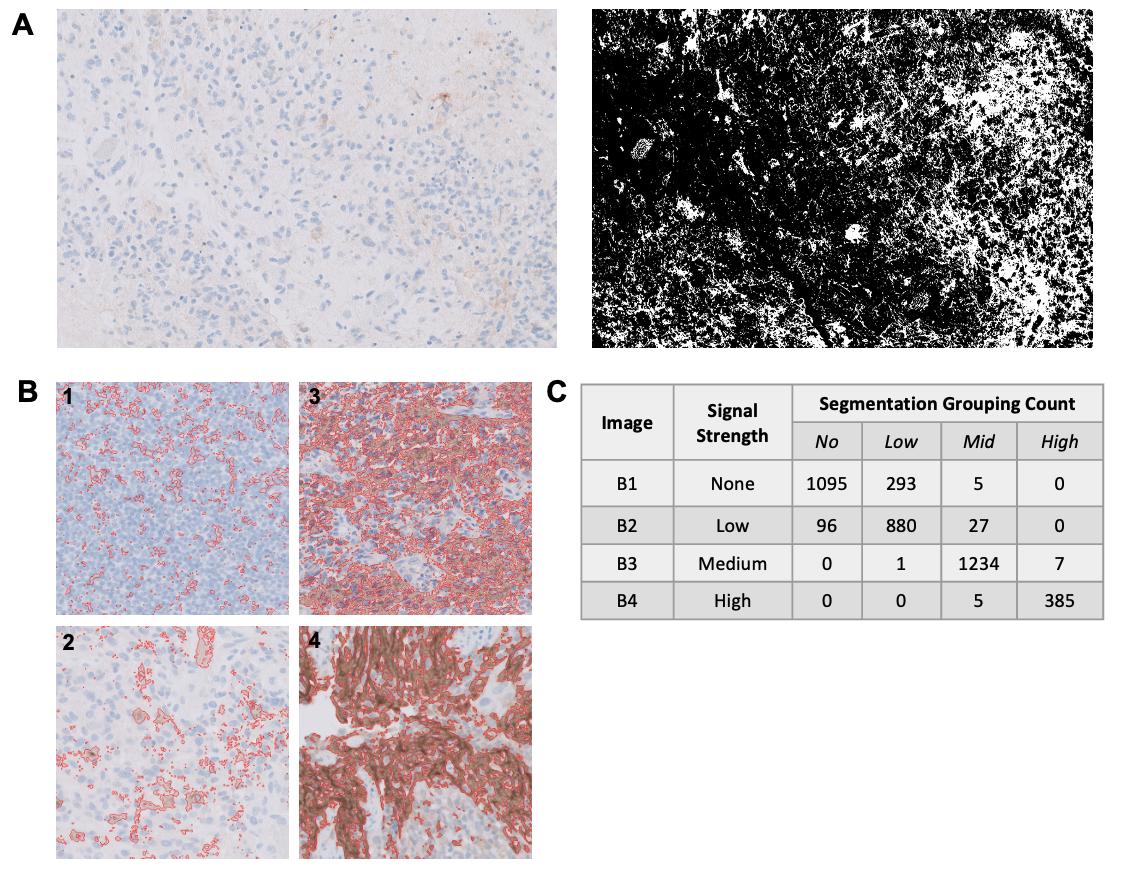
**Supplementary Figure 2: Random Forest model performance of PD-L1 intensity grading.** (A) Thresholding of PD-L1 image with no-low signal highlights tendency of workflow to segment regions of noise that do not capture reality of PD-L1 staining. (B) Representative images of (1) no signal, (2) low signal, (3) medium signal, and (4) high signal intensity for PD-L1 staining were selected and used to train random forest model to classify segments as no, low, mid, or high signal strength using image intensity features. Model performance was assessed using training images as shown in (B) for the relevant signal intensities. (C) Performance of model is highlighted with categorization counts of all segments found in image showing accurate detection of segmentations into representative majority stain.

| **Clinical Feature** | | **Overall** | **OSU** | **UMMC** | **Barretos** | **FLENI** |
| --- | --- | --- | --- | --- | --- | --- |
| *N (%) or mean [range]* | | **N= 581** | **N = 377** | **N = 57** | **N = 94** | **N = 53** |
| Age at Primary Surgery (years) | | 60.64 [20-89] | 61.97 [20-89] | 61.49 [25-80] | 54.13 [29-76] | 61.81 [20-78] |
| Gender | Male | 342 (58.86%) | 219 (58.09%) | 36 (63.16%) | 54 (57.45%) | 33 (62.26%) |
|  | Female | 239 (41.14%) | 158 (41.91%) | 21 (36.84%) | 40 (42.55%) | 20 (37.74%) |
| Race | Native American | 1 (0.17%) | 0 (0%) | 1 (1.75%) | 0 (0%) | 0 (0%) |
|  | Asian | 4 (0.69%) | 4 (1.06%) | 0 (0%) | 0 (0%) | 0 (0%) |
|  | Black or African | 30 (5.16%) | 15 (3.98%) | 15 (26.32%) | 0 (0%) | 0 (0%) |
|  | Caucasian | 467 (80.38%) | 351 (93.10%) | 40 (70.18%) | 76 (80.85%) | 0 (0%) |
|  | More than One Race | 14 (2.41%) | 1 (0.27%) | 0 (0%) | 13 (13.83%) | 0 (0%) |
|  | Unknown | 12 (2.07%) | 6 (1.59%) | 1 (1.75%) | 5 (5.32%) | 0 (0%) |
|  | N/A | 53 (9.12%) | 0 (0%) | 0 (0%) | 0 (0%) | 53 (100%) |
| Ethnicity | Hispanic/Latino | 152 (26.16%) | 5 (1.33%) | N/A | 94 (100%) | 53 (100%) |
|  | Non-Hispanic/Latino | 372 (64.03%) | 372 (98.67%) | N/A | 0 (0%) | 0 (0%) |
|  | N/A | 57 (9.81%) | 0 (0%) | 57 (100%) | 0 (0%) | 0 (0%) |
| Vital Status | Alive | 47 (8.09%) | 23 (6.10%) | 15 (26.32%) | 4 (4.26%) | 5 (9.43%) |
|  | Deceased | 530 (91.22%) | 354 (93.90%) | 42 (73.68%) | 90 (95.74%) | 44 (83.02%) |
| Overall Survival (days) | | 539.6 [31-3685] | 560.4 [52-2787] | 477.5 [45-3685] | 533.8 [94-1471] | 365.9 [31-930] |
| Age at Death (years) | | 62.39 [21-90] | 64.08 [21-90] | N/A | 56.07 [31-76] | 61.61 [46-80] |
| Weight (kg) | | 87.29 [45-214] | 87.29 [45-214] | N/A | N/A | N/A |
| Height (cm) | | 172.5 [147-196] | 172.5 [147-196] | N/A | N/A | N/A |
| BMI | | 29.15 [17.8-66.1] | 29.15 [17.8-66.1] | N/A | N/A | N/A |
| CCI Score | | 3.70 [0-11] | 3.70 [0-11] | N/A | N/A | N/A |
| KPS at Diagnosis | | 79.33 [30-100] | 79.33 [30-100] | N/A | N/A | N/A |
| Lesion Side | Left | 244 (42.0%) | 197 (52.25%) | N/A | 47 (50%) | N/A |
|  | Right | 213 (36.66%) | 170 (45.09%) | N/A | 43 (45.74%) | N/A |
|  | Both | 14 (2.41%) | 10 (2.65%) | N/A | 4 (4.26%) | N/A |
|  | N/A | 110 (18.93%) | 0 (0%) | 57 (100%) | 0 (0%) | 53 (100%) |
| Lesion Location | Frontal | 163 (28.06%) | 142 (37.67%) | N/A | 21 (22.34%) | N/A |
|  | Temporal | 161 (27.71%) | 119 (31.56%) | N/A | 42 (44.68%) | N/A |
|  | Parietal | 97 (16.70%) | 77 (20.42%) | N/A | 20 (21.28%) | N/A |
|  | Occipital | 23 (3.96%) | 22 (5.84%) | N/A | 1 (1.07%) | N/A |
|  | Brain Stem | 14 (2.41%) | 14 (3.71%) | N/A | 0 (0%) | N/A |
|  | Cerebellum | 2 (0.34%) | 2 (0.53%) | N/A | 0 (0%) | N/A |
|  | Mixed | 11 (1.89%) | 1 (0.27%) | N/A | 10 (10.64%) | N/A |
|  | N/A | 110 (18.93%) | 0 (0%) | 57 (100%) | 0 (0%) | 53 (100%) |
| Lesion Size (cm) | | 4.34 [0.1-9.5] | 4.18 [0.1-9.2] | N/A | N/A | N/A |
| Midline Shift | Yes | 141 (24.27%) | 141 (37.40%) | N/A | N/A | N/A |
|  | No | 221 (38.04%) | 221 (58.62%) | N/A | N/A | N/A |
|  | N/A | 219 (37.69%) | 15 (3.98%) | 57 (100%) | 94 (100%) | 53 (100%) |
| ATRX Status | Intact | 427 (73.49%) | 341 (90.45%) | N/A | 86 (91.49%) | N/A |
|  | Loss | 9 (1.55%) | 3 (0.80%) | N/A | 6 (6.38%) | N/A |
|  | N/A | 145 (24.96%) | 33 (8.75%) | 57 (100%) | 2 (2.13%) | 53 (100%) |
| p53 Mutation (>10% of Cells) | Negative | 72 (12.39%) | 72 (19.10%) | N/A | N/A | N/A |
|  | Positive | 256 (44.06%) | 256 (67.90%) | N/A | N/A | N/A |
|  | N/A | 253 (43.55%) | 49 (13.00%) | 57 (100%) | 94 (100%) | 53 (100%) |
| Ki67 (%) | | 31.84 [4-95] | 31.84 [4-95] | N/A | N/A | N/A |
| *EGFR* Amplification | Yes | 173 (29.78%) | 173 (45.89%) | N/A | N/A | N/A |
|  | No | 136 (23.41%) | 136 (36.07%) | N/A | N/A | N/A |
|  | N/A | 272 (46.82%) | 68 (18.04%) | 57 (100%) | 94 (100%) | 53 (100%) |
| *MGMT* Status | Hypermethylated | 189 (32.53%) | 155 (41.11%) | N/A | 34 (36.17%) | N/A |
|  | Unmethylated | 249 (42.86%) | 221 (58.62%) | N/A | 28 (29.79%) | N/A |
|  | N/A | 143 (24.61%) | 1 (0.27%) | 57 (100%) | 32 (34.04%) | 53 (100%) |
| WBC Post-Surgical (x10^9/L) | | 9.78 [2.20-63.77] | 9.96 [2.49-63.77] | 9.76 [2.30-24.80] | 8.72 [2.20-18.60] | 10.57 [5.10-20.50] |
| Neutrophils Post-Surgical (x10^9/L) | | 7.21 [0.21-22.82] | 7.37 [1.00-21.50] | 7.26 [0.21-22.82] | 6.02 [0.97-16.00] | 8.53 [2.35-18.25] |
| Lymphocytes Post-Surgical (x10^9/L) | | 1.65 [0.07-54.2] | 1.65 [0.08-54.2] | 1.59 [0.07-4.68] | 1.80 [0.12-5.60] | 1.38 [0.38-3.54] |
| Neutrophil: Lymphocyte Ratio Post-Surgical | | 7.51 [0.06-137.90] | 8.00 [0.06-115.13] | 6.84 [0.85-30.67] | 5.53 [1.01-137.90] | 9.19 [1.12-32] |
| Platelets Post-Surgical (x10^9/L) | | 236.5 [43-593] | 231.6 [64-593] | 246.2 [43-522] | 247.9 [84-467] | N/A |
| Steroids Post-Surgical (mg/day) | | 2.84 [0-24] | 2.67 [0-24] | 0.31 [0-2] | N/A | 6.87 [0-16] |
| Radiation Dose (Gy) | | 55.59 [5.34-75] | 55.82 [5.34-75] | N/A | 54.66 [25-66] | N/A |
| Radiation Fractions | | 26.8 [1-50] | 26.74 [1-50] | N/A | 27.07 [5-33] | N/A |
| ChemoRT Treatment Time (Days) | | 42.2 [0-336] | 38.14 [0-90] | N/A | 62.09 [0-336] | N/A |
| Adjuvant Temozolomide Dose (mg) | | 238.1 [0-500] | 238.1 [0-500] | N/A | N/A | N/A |
| Adjuvant Temozolomide Cycles | | 3.00 [0-19] | 3.072 [0-19] | N/A | 2.70 [1-12] | N/A |
| Time to Enhancement (days) | | 278.6 [41-2037] | 278.6 [41-2037] | N/A | N/A | N/A |
| Enhancement Status | Recurrent | 241 (41.48%) | 241 (63.94%) | N/A | N/A | N/A |
|  | Reactive | 104 (17.90%) | 104 (27.59%) | N/A | N/A | N/A |
|  | Stable | 32 (5.51%) | 32 (8.49%) | N/A | N/A | N/A |
|  | N/A | 204 (35.11%) | 0 (0%) | 57 (100%) | 94 (100%) | 53 (100%) |

**Supplementary Table 2: Summarization of collected clinical features across sites.** N/A represents uncollected

| Feature | Beta | HR (95% CI) | Wald Test | | P-value |
| --- | --- | --- | --- | --- | --- |
| *Age at Surgery* | 0.01944 | 1.02 (1.011-1.028) | 21 | 4.20E-06* | |
| *Gender* | -0.01552 | 0.9846 (0.8232-1.178) | 0.03 | 0.87 | |
| *Race* | -0.002198 | 0.9978 (0.9908-1.005) | 0.37 | 0.54 | |
| *Ethnicity* | 0.1583 | 1.172 (0.9444-1.453) | 2.1 | 0.15 | |
| *Weight* | 0.002215 | 1.002 (0.9979-1.007) | 0.99 | 0.32 | |
| *Height* | 0.004985 | 1.005 (0.9954-1.015) | 1 | 0.31 | |
| *BMI* | 0.004967 | 1.005 (0.9892-1.021) | 0.38 | 0.54 | |
| *KPS* | -0.02359 | 0.9767 (0.9682-0.9853) | 28 | 1.20E-07* | |
| *CCI Score* | 0.05545 | 1.057 (1.013-1.103) | 6.5 | 0.011* | |
| *Lesion Side* | 0.03169 | 1.032 (0.868-1.228) | 0.13 | 0.72 | |
| *Lesion Lobe* | -0.01187 | 0.9882 (0.913-1.07) | 0.09 | 0.77 | |
| *ATRX* | 0.1074 | 1.113 (0.5269-2.353) | 0.08 | 0.78 | |
| *p53* | -0.1035 | 0.9017 (0.6851-1.187) | 0.55 | 0.46 | |
| *Ki67* | 0.00311 | 1.003 (0.9966-1.01) | 0.88 | 0.35 | |
| *EGFR* | -0.2294 | 0.795 (0.6275-1.007) | 3.6 | 0.057 | |
| *MGMT* | -0.4272 | 0.6523 (0.533-0.7983) | 17 | 3.40E-05* | |
| *Lesion Size* | 0.01855 | 1.019 (0.9616-1.079) | 0.4 | 0.53 | |
| *Midline Shift* | -0.1176 | 0.8891 (0.7115-1.111) | 1.1 | 0.3 | |
| *WBC* | 0.02951 | 1.03 (1.013-1.047) | 13 | 0.00036* | |
| *Platelets* | 0.000202 | 1 (0.9991-1.001) | 0.13 | 0.72 | |
| *Neutrophils* | 0.04722 | 1.048 (1.023-1.074) | 14 | 0.00015* | |
| *Lymphocytes* | 0.01214 | 1.012 (0.9767-1.049) | 0.44 | 0.51 | |
| *NLR* | 0.01261 | 1.013 (1.005-1.021) | 9.3 | 0.0023* | |
| *Steroids* | 0.02151 | 1.022 (0.9942-1.05) | 2.4 | 0.12 | |
| *Radiation Dose* | -0.05025 | 0.951 (0.9413-0.9608) | 92 | 9.80E-22* | |
| *Radiation Fractions* | -0.05064 | 0.9506 (0.9373-0.9641) | 50 | 1.70E-12* | |
| *Radiation Time* | -0.003487 | 0.9965 (0.9931-1) | 3.9 | 0.049* | |
| *TMZ Cycles* | -0.1612 | 0.8511 (0.8216-0.8816) | 80 | 3.30E-19* | |
| *TMZ Dose* | -0.00238 | 0.9976 (0.9969-0.9984) | 41 | 1.80E-10* | |
| *Center* | -0.05162 | 0.9497 (0.8576-1.052) | 0.98 | 0.32 | |

**Supplementary Table 3. Univariate cox model results.** Influence of clinical features to overall survival was evaluated using univariate cox regression. Features indirectly related to OS were omitted. Significant features are marked by asterisk.

| **Feature** | **Beta** | **HR (95% CI)** | **P-value** |
| --- | --- | --- | --- |
| **Age at Surgery** | 0.01652 | 1.017 (1.001-1.032) | 0.036* |
| **KPS** | -0.01879 | 0.9814 (0.9716-0.9913) | 0.00025* |
| **CCI Score** | -0.02579 | 0.9745 (0.9106-1.043) | 0.46 |
| **MGMT** | -0.4454 | 0.6406 (0.4879-0.841) | 0.0013* |
| **WBC** | 0.02298 | 1.023 (1.001-1.046) | 0.045* |
| **Radiation Dose** | -0.03813 | 0.9626 (0.9432-0.9823) | 0.00023* |
| **Radiation Time** | 0.00719 | 1.007 (0.9924-1.022) | 0.34 |
| **TMZ Cycles** | -0.1971 | 0.8211 (0.7737-0.8714) | 8.10E-11* |
| **TMZ Dose** | 0.0004779 | 1 (0.9994-1.002) | 0.4 |

**Supplementary Table 4. Multivariate cox model results.** Influence of clinical features to OS was evaluated using multivariate cox regression from features in table 3. Significant features are marked by asterisk.

**
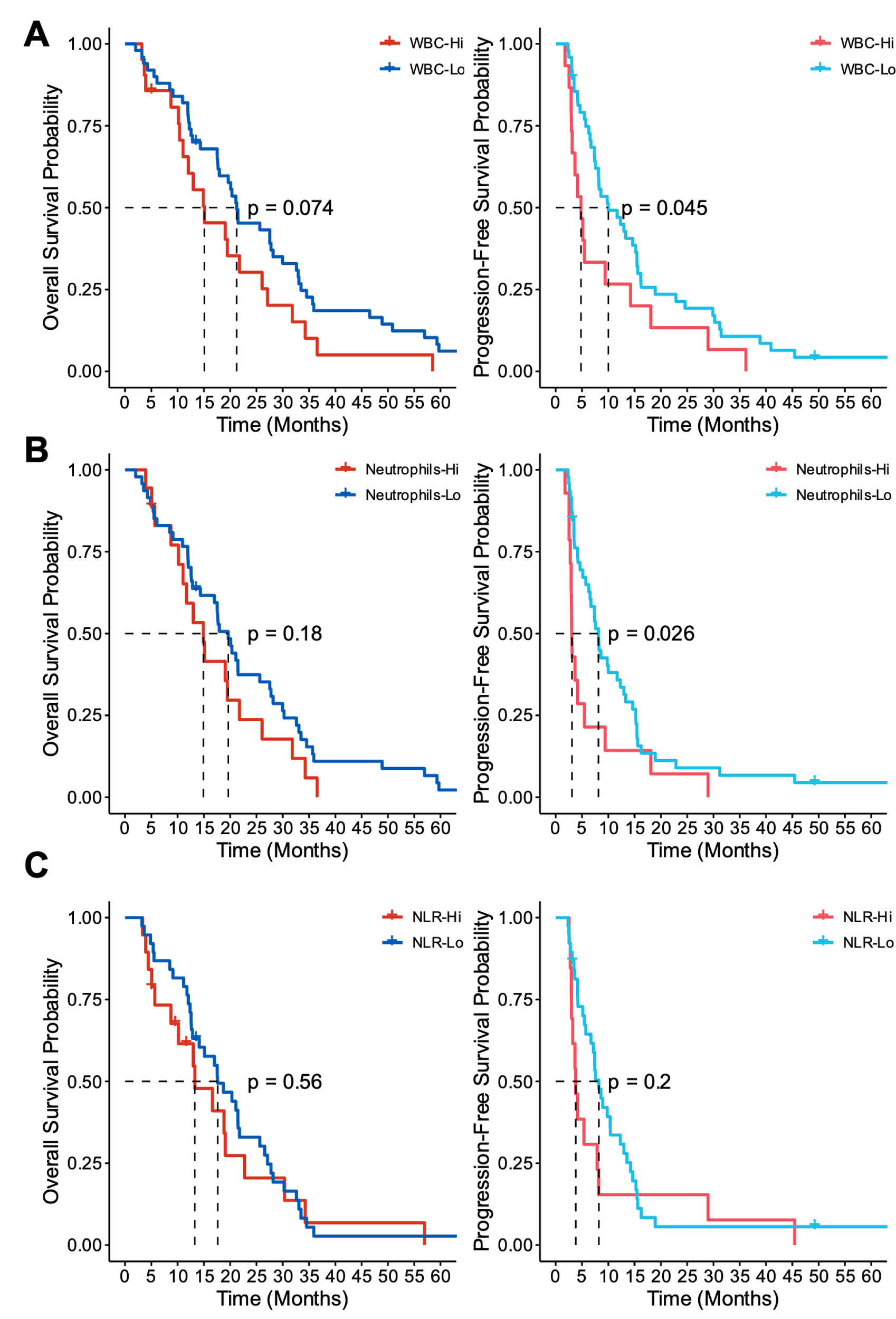
**

**Supplementary Figure 3: KM curve survival outcomes based upon CBCs in patients fully tapered off steroids.** OS (left) and PFS (right) of patients in upper 25% [red] and lower 25% [blue] of cases stratified based on (A) WBC count, (B) Neutrophil count, and (C) Neutrophil-to-Lymphocyte ratio. All CBCs were collected prior to initiating ChemoRT as routine baseline by neuro-oncology. Cut-offs as delineated in Supplementary Table 1 were used. All represented patients had completed steroid taper at CBC draw.
